# An Association of Human Papillomaviruses Low Risk and High Risk Subtypes with Skin Tag 

**Published:** 2012

**Authors:** Fakhrozaman Pezeshkpoor, Amir Hossein Jafarian, Kiarash Ghazvini, Mohammad Javad Yazdanpanah, Ali Sadeghian, Habiballah Esmaili, Maryam Karrabi, Fatemeh Rohani, Bahareh Joushan

**Affiliations:** 1 * Department of Dermatology, Mashhad University of Medical Sciences, Mashhad, Iran*; 2 * Department of Pathology, Mashhad University of Medical Sciences, Mashhad, Iran*; 3 * Department of Microbiology, Mashhad University of Medical Sciences, Mashhad, Iran*; 4 * Community Health and Statistic Department, Mashhad University of Medical Sciences, Mashhad, Iran*; 5 * Medical Student, Mashhad, University of Medical Sciences, Mashhad, Iran*; 6 * Endodontist, Mashhad, Iran*; 7 * Dentist, Mashhad, Iran*

**Keywords:** HPV, PCR, Skin Tag

## Abstract

**Objective(s):**

Human papillomavirus (HPV) infections are related to the genesis of various benign lesions and some malignant tumors, but no clear relationship has been identified so far between the subtypes of HPV and skin tag.

**Materials and Methods:**

The present case-control study was designed to detect the existence of low risk and high risk HPV types in lesions of 50 patients with skin tag (case group) and normal skin around the melanocytic nevus of 30 patients (control group), using PCR.

**Results:**

All of the samples were negative for HPV subtypes, except two samples in control group which were positive for high risk HPV. There was no significant relationship between the HPV subtypes and skin tag.

**Conclusion:**

There is no association between skin tag and low risk and high risk human papillomaviruses.

## Introduction

Skin tag is a common disease in the societies which has unknown causes. It usually involves flexural area such as axilla, groin, and neck and in rare cases genital and anus area. However, the most common site of involvement is neck (1).

Skin tag is more common in elderly people, especially in the menopause women and it could be accompanied with seborrheic keratosis, melanocytic nevus and neurofibroma. Histological findings show a normal or hyperplastic epidermis overlying a fibrovascular connective tissue core. However, diagnosis is made mainly clinical; and histology examination is only for confirmation. Different treatment protocols are available for skin tag such as cryotherapy (using liquid nitrogen -196 centigrade degree), cutter, and surgery (2, 3).

HPV or human papillomavirus is a 50-55 nm diameters DNA virus with which involves squamous epithelium and causes cell proliferation. HPV is contagious and transmission is possible. Infected person will have skin problems after an incubation period. Study results have shown a relationship between HPV and skin tag, which will imply the viral cause for skin tag (4, 5). There is no reported study on the relationship between HPV and skin tag in Iran. PCR is a useful and effective method for detecting viral DNA in tissue or blood, which is more precise than other diagnostic tests like culture and serology (4). Moreover, PCR is used to determine virus type and to differentiate between low risk and high risk viruses. In the present study, PCR was used to detect low risk and high risk HPV to evaluate the relationship between these types of HPV and skin tag.

## Materials and Methods

This study was performed on skin tag of patients with clinical and pathological diagnosis of skin tag in axilla, groin and neck areas. Fifty patients with skin tag and thirty specimens from normal skin near melanocitic nevus were prospectively included in the present study. After reconfirmation of histological diagnosis, the specimens were cut and chapped. DNA was then extracted from these tissue samples using commercial DNA isolation kit from tissue (PrimePrep^TM ^Genomic DNA Isolation Kit from Tissue) according to the manufacturer’s instructions (GENETBIO, www.genetbio.co.kr).

The quality of the DNA sample and the absence of PCR inhibitors were checked in all samples by the amplification of part of the human b-actin gene: (forward: 5'- TCCTGTGGCCATCCACAACT-3' and reverse:

5'- GAAGCATTTGCGGTGGACCAT-3') 300 bp target as described before (6).

 The presence of HPV low risk DNA (type 6, 11, 42, 53, 54, 66, 68) was tested by PCR Kit (GenePak DNA PCR test, Isogene Lab Ltd, Russia) which could detect several type of low risk HPV (type 6, 11, 42, 53, 54, 66, 68) according to the manufacturer’s instructions*. *Then the presence of high risk HPV was also investigated by PCR amplification using general-purpose HPV primers (forward: 5'-TTTGTTACTGTGGTAGATACTAC-3' and reverse: 

5'-GAAAAATAAACTGTAAATCATATTC-3') which amplify conserved sequences in the HPV-L1 region as previously described (6-9). The amplification cycles comprise 1 min at 94 °C, 2 min at 40 °C, and 1.5 min at 72 °C, with the final extension step prolonged to 4 min to ensure complete amplification of the target in a techne gradient thermal cycler (TC-5000 gradient thermal cycler, Techne, UK). Positive and negative controls were included in each batch of amplifications. PCR products were analyzed in 1.5% agarose gel (Sigma) and were visualized by etidiumbromide (ETBr) under UV light. Visualizing the 450 bp fragment and 149 bp fragment was interpreted as positive result for low risk HPV and high risk HPV respectively. Precautions to avoid cross-contamination and false-positive results were taken in every assay (10). Any positive high risk HPV specimens were subjected to subtyping by performing PCR as described earlier (6). Statistical analysis was done by SPSS version 13 software. The Chi-Square test, Mann–Whitney test, and Fisher exact tests were also used. The results were considered significant when the *P*-value was <0.05.

## Results

**Figure 1:**
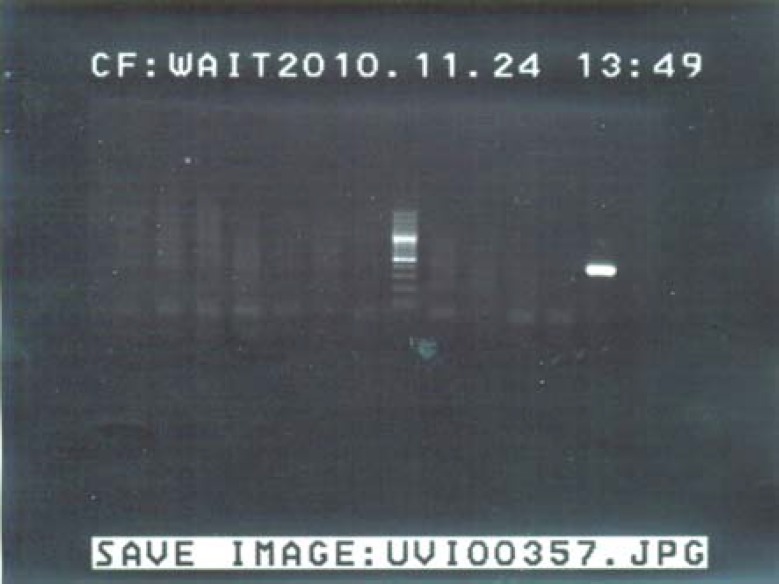
Gel electrophoresis PCR with low risk HPV. Line 1 is positive control (positive band is amplified by HPV DNA of 149 base pairs), line 2 is negative control, line 6 is weight marker (100 bp ladder). Other lines are negative samples.

**Figure 2:**
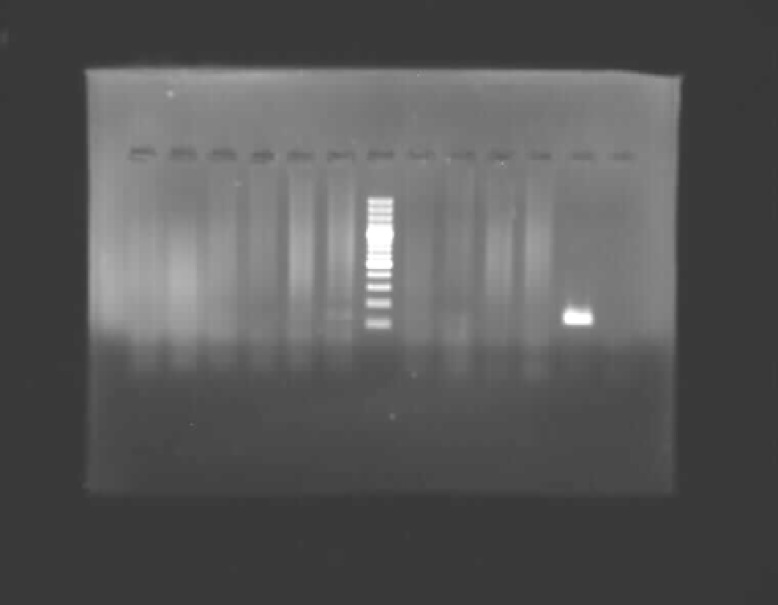
Gel electrophoresis PCR with high risk HPV. Line 1 is positive control (positive band is amplified by HPV DNA of 450 base pairs), line 2 is negative control, line 6 is weight marker 100 bp ladder. Other lines are negative samples.

In the present study, the case group included fifty patients with average age of forty five, and thirty patients with the average age of thirty nine were included in the control group. Twenty nine women and twenty one men were in case group and twenty women and ten men were in control group. Samples in patient group were taken from following areas: twenty six samples (52%) neck, thirteen samples (26%) axilla and eleven samples (22%) groin. In control group twenty samples (66.7%) were taken from face, six samples (20%) from trunk and four samples (13.3%) from arm. Chi-Square test showed no significant difference regarding the sex of the case and control group (*P*= 0298). Mann-Whitney test showed no significant difference regarding the age of case and control group (*P*= 0.14). High risk HPV was detected in only two samples which belonged to the control group (Figure 1); and there was no high risk HPV in case group. Chi-Square test showed no significant difference for high risk virus type between two groups (*P*= 0.138) (Table 1). Moreover, there was no significant difference for high risk HPV between sex groups (*P*= 0.628). All the samples in both groups were negative for low risk HPV (Figure 2). Similarly Chi-Square test showed no significant difference for low risk virus type between two groups, or any significant difference for low risk HPV between sex groups and age groups.

## Discussion

A high number of HPV infection exists in tropical parts of world, therefore there is the possibility that continuous contact with sun ray reinforces HPV infection. Ultra violet light has many effects on dermis immunity including reducing the number of Langerhas cells in epidermis and decreasing their antigen production ability, increasing the production of IL-10 and prostaglandin E2 by keratonocytes, and increasing the serum level of IL-4 (11). It may be concluded that ultra violet light suppress derm immunity with reducing activity of TH1 cells (12). This will be an ideal situation for human papillomavirus. 

Skin tag or fibroepitheial polyps are developed in areas of skin which are prone to rubbing. These areas are probably entrance routes of virus. HPV DNA and mechanical rubbing are cofactors and have role in pathogenesis of skin tag (12). In the present study no low risk HPV was detected in any of study groups. The high risk HPV was detected 

**Table1:** Comparing case and control groups with low risk and high risk HPV

HPV	Low risk					High risk				
Group	Positive	%	Negative	%	*P* value	Positive	%	Negative	%	*P-* value
Case	0	0	50	100	0	0	0	50	100	0.138
Control	0	0	30	100		2	6.6	28	93.4	
Total	0	0	80	100		2	2.5	97.5	100	

in 2 samples of control group, concluding that there is no significant relation between HPV and skin tag. The negative results of HPV PCR could be due to one of the following reasons:

- The lack of any link between HPV and skin tag.

- Technical errors

Since in the present study, sensitive and specific methods were used to detect the tissue HPVs, therefore the negative results cannot be due to technical errors because:

- PCR method used in this study is sensitive.

- A positive control in all HPV PCRs was used and it showed the expected fragment size using DNA size marker. Also, the negative control was negative by the HPV PCR.

The quality of each DNA sample was verified by amplification of portions of human β-actin gene. Successful amplification of the β-actin gene fragment indicated the integrity of the DNA samples and the absence of PCR inhibitors (6-8). 

In Gopta study, 48.6% of samples have shown HPV with genome sequence 11, 6, while HPV was not detected in any samples taken from areas around the skin tag (control group) (13). in Dianzy study HPV 11,6 was positive in 88% of patients while it was negative in tissue samples from healthy people and samples from around skin tag (4). In a study done by Sallam *et al*, HPV DNA was positive in 77% of skin tags and had significant difference with samples taken from around skin tag (53% positive) and control group (20% positive). Therefore, they concluded that HPV has an important role on the pathogenesis and progress of skin tag (5). Although all of the above-mentioned studies have emphasis the relation between HPV and skin tag, however, the present study’s results are not compliance with them.

In the present study PCR was done after pathology confirmation and definite diagnosis of skin tag, but in the reported studies pathology confirmation have not been done and diagnosis was made only on the basis of clinical appearance. Control group could also be a reason for incompliance. In the present study, healthy skin around the melanocitic nevus was used as control samples, while in other studies the healthy skin around skin tag was considered as control group. Since HPV infection could be subclinical, it could justify the positive results in our control group. 

Considering the fact that HPV proliferates in keratinicytes of basal layer of dermis, and is transmitted to cells of upper epiderm layer, it is usually associated with the epidermal proliferations such as wart, while skin tag is a benign connective tissue stromal tumor (1). This fact can explain the lack of any relation between HPV and skin tag.

There was no significant relationship between low risk/high risk virus infection, age and gender of patients, which is in agreement with reported studies results.

Based on the results of the present study, no precautions are necessary when contacting skin tags like those considered when contacting wart or premalignant diseases. Considering the fact that high risk HPV was positive around melanocitic nevus, the researchers suggest a further study be conducted on the relation between melanocitic nevus and high risk HPV. 

## Conclusion

According this study results, there is no association between skin tag and low risk and high risk human papillomaviruses.
